# Enhancing plasticity to treat depression and other central nervous system diseases using event-driven pharmacology

**DOI:** 10.1177/02698811261456206

**Published:** 2026-07-13

**Authors:** Todd D. Gould, Sanjay J. Mathew, Maurizio Fava, Anantha Shekhar

**Affiliations:** 1 Departments of Psychiatry, Pharmacology-Physiology and Drug Discovery, and Neurobiology, University of Maryland School of Medicine, Baltimore, USA; 2 Veterans Affairs Maryland Health Care System, Baltimore, USA; 3 Department of Psychiatry and Behavioral Sciences, Naresh K. Vashisht College of Medicine, Texas A&M University, Bryan, USA; 4 Department of Psychiatry, Massachusetts General Hospital, Boston, USA; 5 School of Medicine, University of Pittsburgh, PA, USA

**Keywords:** metaplasticity, depression, ketamine, plastogen, neuroplastogen, metaplastogen

## Abstract

The occupancy-driven pharmacology model in psychopharmacology has guided drug discovery and development for decades, whereby the goal is stable receptor occupancy over an extended period with a direct relationship of drug exposure to pharmacodynamic and therapeutic response. Event-driven pharmacology (EDP) is a concept where the acute pharmacodynamic actions of a plastogen, a transient binding event, drive changes resulting in sustained pharmacodynamic effects that greatly outlast the time frame of drug exposure. Such plastogens may be neuroplastogens producing a measurable change in neuroplasticity without inducing subjective mental states or psychoplastogens exerting neuroplastic effects in addition to causing dissociation, hallucinations, or psychotomimetic symptoms. Within neuropsychiatry, plastogens have been shown to induce long-lasting neural plasticity and persistently activate synaptic machinery that primes synapses to respond to subsequent stimuli (metaplasticity), often following either a single dose or repeated administration of intermittent doses. Developing rapid-acting plastogens to adhere to a traditional target occupancy dose optimization model that achieves consistent drug target occupancy over an extended period of time may paradoxically exert few or no durable benefits while increasing side effect burden. We review evidence supporting the concept that plastogens, including the rapid-acting antidepressant ketamine and classical psychedelics, operate within the EDP model. With the emergence of EDP, new methods for drug development, clinical dosing, and biomarker adoption will need to be developed to account for distinctive pharmacological actions. Rapid-onset plastogens, rationally administered, have profound potential in the treatment of depression and many other neurological and psychiatric diseases characterized by synaptic dysfunction.

## Introduction

**Figure 1.**
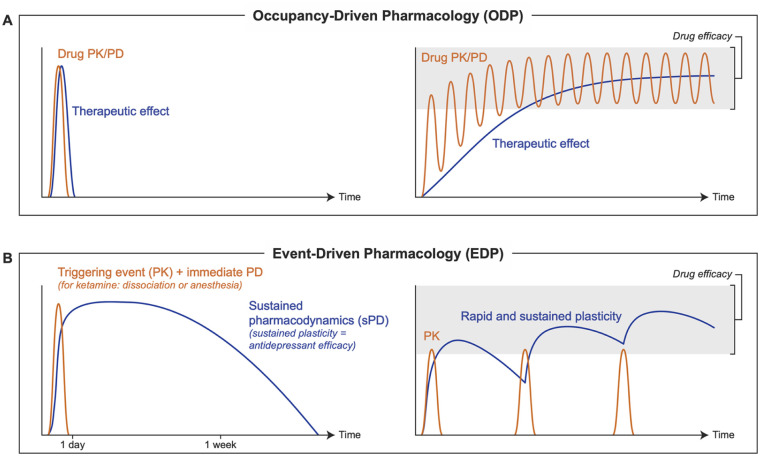
Pharmacokinetic and therapeutic effects in ODP versus EDP. In the traditional ODP approach to drug pharmacokinetics, pharmacological response is directly dependent on drug exposure. As the drug or any biologically active metabolites are eliminated, the response diminishes. In ODP (panel a), the sustained therapeutic effect is achieved by repeated dosing targeting stable receptor occupancy. In EDP (panel b), the sustained plasticity and resultant therapeutic benefits outlast the drug’s exposure and can be induced by a single administration or repeated intermittent administration and no sustained receptor occupancy. Repeated intermittent dosing in EDP can be used to sustain the therapeutic effect while minimizing drug exposure and associated side effects. In contrast, too frequent dosing results in physiological insensitivity or engagement of compensatory homeostatic mechanisms to prevent further plasticity. PD: pharmacodynamics; PK: pharmacokinetics.

Historically, therapeutic drug discovery and development in psychiatry have been focused on the receptor occupancy-driven pharmacology (ODP) model, which is based on optimizing molecules that exert modulation of protein function (typically neurotransmitter receptors) through continuous and chronically sustained stoichiometric drug interactions with the binding site (Figure 1(a); Table 1). In clinical development, the optimal biological dose, defined as a dose that sufficiently binds a drug target or maintains a target plasma concentration without undue side effect burden, has been informed by the use of ODP (Schmidt, 1988). In this method, the efficacy of the drug is reliant on sufficient and consistent occupancy of the target binding site in the target organ, dependent upon complexities of drug distribution and exposure (Louizos et al., 2014). Sustained systemic drug concentrations required to maintain sufficient levels for target engagement commonly result in side effects and off-target adverse effects, resulting in a decreased dose range to limit undesirable effects while still achieving sufficient and sustained target engagement.

**Table 1.** Comparison of occupancy-driven pharmacology (ODP) versus event-driven pharmacology (EDP) models.

Model	Dose	Dosing frequency
ODP	Maximum tolerated dose defined as within an acceptable side effect and toxicity window	Stable receptor occupancy over an extended time of weeks, months, and longer
EDP	May exhibit a *U*-shaped dose response where higher doses overstimulate (either via intensity or time of exposure) the system, independent of side effects, resulting in limited sustained response	Transient receptor occupancy, followed by complete drug elimination. Intermittent dosing allows for persistent therapeutic effects following target engagement

Ketamine and other rapid-acting molecules that act on plasticity pathways exhibit a nonconventional relationship between exposure, target occupancy, and therapeutic effect (i.e., a disconnect between pharmacokinetics and pharmacodynamics), informing the need for a different construct of pharmacology (Figure 1). Event-driven pharmacology (EDP) has emerged as a model of pharmacology with therapeutic potential in a variety of fields and requires revisiting widely accepted clinical pharmacology principles (Cromm and Crews, 2017; Lai and Crews, 2017). In this pharmacology model, a transient interaction of the drug with its target initiates an “event,” which leads to sustained changes. In contrast to ODP, EDP does not include the goal of continuous or chronically sustained drug exposures at the target organ but rather short-term drug exposure and interaction with the initial target. The result is event-driven pharmacodynamic effects that greatly outlast the pharmacokinetics of the drug, its biologically active metabolites, and their activity at the initial target binding sites (Figure 1(b); Table 1). The term “EDP” was initially described in the context of small molecule protein degradation strategies (Cromm and Crews, 2017), which we have adapted to apply toward targeting plasticity mechanisms where drugs may produce sustained therapeutic actions that extend long past direct target engagement. Emerging classes of drugs, such as plastogens, may exert their therapeutic effects in the central nervous system (CNS) via EDP.

## Neuroplasticity and the role of plastogens

Synaptic dysfunction and disruption of normal network activity are common underlying pathologies among neurodevelopmental, neuropsychiatric, and neurodegenerative disorders (Bliss et al., 2014; Manji and Duman, 2001). Depression in particular is associated with reduced synaptic density and impaired cortical functional connectivity (Abdallah and Krystal, 2020; Krystal et al., 2023). Neuroplasticity is a broad term that describes diverse processes by which neurons adapt and respond to stimuli. Neuroplasticity includes long-term potentiation (LTP) or long-term depression (LTD) of synaptic strength, depending on the changes in synaptic strength observed, which are achieved via distinct engagement of multiple membrane glutamate receptors and resultant activation of intracellular mechanisms that change the function and localization of receptors (Nicoll, 2017). Plastogens are defined as agents that induce neuroplasticity and can include rapid-onset agents, such as neuroplastogens, psychoplastogens, and metaplastogens (Brown and Gould, 2024; Cooper et al., 2023; Ly et al., 2021; Olson, 2018; Vargas et al., 2021; Warner-Schmidt et al., 2024). A neuroplastogen is a compound that produces a change in neuroplasticity without inducing subjective mental states, whereas a psychoplastogen such as ketamine or psilocybin produces a change in neuroplasticity while also inducing subjective mental states, such as dissociation, hallucinations, and/or psychotomimetic symptoms (Brown and Gould, 2024; Cooper et al., 2023). Plastogens likely operate within the pharmacological category of EDP, whereby the administration of these compounds facilitates a triggering event that generates measurable changes in neuroplasticity, or the future potential for plasticity (metaplasticity), that far outlast their elimination.

Metaplasticity is the process by which a triggering event, which may be pharmacological or physiological, prompts changes in cellular or synaptic properties that by themselves do not change synaptic strength but impact the ability of synapses for plasticity, or a network of synapses, to be readily activated in response to future stimulation forming greater, or longer, retainment of synaptic changes (Table 1; Abraham, 2008). Several plastogens have been shown to induce metaplasticity, often following either a single dose or repeated administration of intermittently spaced doses (Brown and Gould, 2024).

Neuroplasticity-inducing stimuli are documented to have well-defined characteristics that include both intensity and the temporal spacing of stimuli. For example, while there are standard stimulation conditions whereby LTP is generated, a greater magnitude of or additional stimuli pulses beyond these conditions do not result in further synaptic potentiation and, in some cases, have no effect or a reversal of effects (Abraham and Huggett, 1997; Christie et al., 1995). Thus, higher levels (either of magnitude or frequency) of stimulation can result in a lower increase in synaptic strength than lower levels of stimulation, thereby generating a *U*-shaped response curve.

Plastogens that promote the ability of neurons to modify in response to stimuli or stimulate changes to their structure and function have the potential to treat a variety of brain diseases (Krystal et al., 2009; Manji and Duman, 2001; Taoufik et al., 2018). Research in major depressive disorder (MDD) is uncovering pharmacologically diverse compounds that have rapid antidepressant effects. Such findings are resulting in a paradigm shift in the approach to depression treatment, whereby the expectation is positive treatment effects in hours or days rather than weeks or months (Gould et al., 2019). A large amount of the evidence supporting the sustained effects of rapid-onset plastogens is based on ketamine, and this knowledge has driven the field to further investigate rapid-acting antidepressant mechanisms (Riggs and Gould, 2021). Ketamine is an open-channel blocker of the *N*-methyl-D-aspartate receptor (NMDAR), an ionotropic glutamate receptor, and studies have suggested that NMDAR antagonism at the phencyclidine binding site is responsible for the dissociative and psychotomimetic effects of ketamine (Ballard and Zarate, 2020; Krystal et al., 1994; Zanos et al., 2018). In 2000, ketamine was first reported to have rapid antidepressant effects at subanesthetic doses (Berman et al., 2000). The antidepressant effects of a single dose of subanesthetic ketamine in individuals with treatment-resistant depression can emerge about 2–4 hours after intravenous administration and typically abate within about 1 week, which is well after the parent compound clears from the body (Aan Het Rot et al., 2012; Zarate et al., 2006). Repeated intermittent administration typically leads to greater and longer sustained effects (Chen et al., 2021; Kryst et al., 2020; Le et al., 2026; Nikolin et al., 2023; Phillips et al., 2019; Shiroma et al., 2020; Zheng et al., 2018). In contrast to ODP predictions, preclinical studies indicate that antidepressant-like behavioral effects following administration of ketamine follow a *U*-shaped dose response, with subanesthetic doses being effective but anesthetic doses not exerting an effect (Li et al., 2010; Kim and Monteggia, 2020; Zanos et al., 2016, 2023).

The mechanisms underlying the sustained effects of ketamine are not fully understood; however, treatment with ketamine is associated with numerous changes in structural and physiological plasticity (Liao et al., 2025). Subanesthetic, but not anesthetic, doses of ketamine increase the activity of neuroplasticity-associated cellular signaling pathways (Kim and Monteggia, 2020; Li et al., 2010; Zanos and Gould, 2018). Administration of ketamine to rodents has been reported to enhance the capacity for synaptic plasticity (i.e., exert metaplasticity) at time points long after drug elimination (Aleksandrova et al., 2020; Burgdorf et al., 2013; Graef et al., 2015; Le et al., 2026; Widman et al., 2018), and consistent with canonical LTP mechanisms, these effects require NMDAR activity (Zanos et al., 2023). The sustained antidepressant effects appear to be independent of ODP, as most standard effective ketamine treatment paradigms include treatments separated by several days (McIntyre et al., 2021; Wajs et al., 2020). Repeated daily ketamine administration over 3 days did not result in an improved antidepressant response compared with placebo (Pattanaseri et al., 2024), though we note that this research study did not include an intermittent treatment group as a comparison. Instead, the rapid antidepressant effects are better explained through EDP, where the effects are hypothesized to be achieved by direct effects on plasticity (Gould et al., 2019) or a shift in the threshold for induction of synaptic plasticity through metaplasticity (Brown et al., 2025b). Though rapid-onset plastogens may have different direct pharmacological actions, they appear to converge on similar molecular mediators that result in altering capacity for synaptic plasticity (Brown and Gould, 2024). Like ketamine, serotonergic psychedelics, such as psilocybin, have demonstrated rapid and long-lasting antidepressant effects following 1 or 2 doses (Carhart-Harris and Goodwin, 2017; Griffiths et al., 2016; Goodwin et al., 2022; Raison et al., 2023). Ketamine and serotonergic psychedelics in preclinical and clinical studies have shown many similarities in their therapeutic effects (Dai et al., 2023; De Gregorio et al., 2021; Ly et al., 2018; Nardou et al., 2023). Synaptic plasticity mediated by psychedelics (specifically, psychedelics that target serotonin (5-HT) receptors: lysergic acid diethylamide, N,N-dimethyltryptamine, and psilocybin) is not fully understood; however, multiple studies investigating the therapeutic application of psychedelics have found evidence supporting 5-HT receptor–dependent rapid enhancement of synaptic plasticity (De la Fuente Revenga et al., 2021; Ly et al., 2021), shown to promote dendritic branching and increased spine and synapse number in cultured neurons and in vivo (Liao et al., 2025; Ly et al., 2018), or activation of metaplasticity pathways (Nardou et al., 2023) similar to prior findings with ketamine. Considering the limited dosing schedule found to be effective with serotonergic psychedelics, the ODP model does not sufficiently describe the rapid and sustained therapeutic effects seen with psychedelics (Nichols et al., 2017).

While evidence has linked the antidepressant mechanism of ketamine to NMDAR inhibition (Autry et al., 2011; Krystal et al., 2024; Li et al., 2010; Miller et al., 2014; Yang et al., 2018), this is an active area of research. Numerous studies have found that other NMDAR antagonists that bind to the same channel pore site on the NMDAR, like ketamine and others with NMDAR subtype specificity (e.g., GluN2B antagonists), do not consistently induce the same rapid-acting and sustained antidepressant effects as seen with ketamine in clinical studies (Gould et al., 2019; Hsu et al., 2022; Newport et al., 2015; Sanacora et al., 2017). Evidence indicates that high affinity for the NMDAR is associated with the dissociative effects of ketamine and that the mechanism responsible for the rapid-acting antidepressant effect may be independent of NMDAR inhibition (Zanos et al., 2018). For example, the (*2R,6R*)-hydroxynorketamine (HNK) metabolite of ketamine rapidly exerts ketamine-like effects on plasticity in preclinical models without NMDAR inhibition (Zanos et al., 2016) and without dissociative side effects in humans (Raja et al., 2024). Additionally, (*2R,6R*)-HNK exerts metaplasticity actions in preclinical model systems (Brown et al., 2025a). Separate evidence suggests that ketamine acts in part via opioid receptors to exert antidepressant actions (Williams et al., 2018), and recent preclinical evidence has implicated adenosine signaling (Yue et al., 2026).

Positive allosteric modulators (PAMs) of the NMDAR enhance the function of NMDARs, leading acutely to enhanced plasticity and to increased metaplasticity processes, as evidenced by increased capacity for LTP at long time points following administration (Burgdorf et al., 2013, 2015, 2022; Donello et al., 2025; Khan et al., 2018). Unlike ketamine and psychedelic compounds, this drug class appears to exert their effects devoid of psychotomimetic and dissociative symptoms (Burgdorf et al., 2015; Kato and Duman, 2020). Preclinical studies have identified that the NMDAR PAM rapastinel produces antidepressant-like effects for up to 1 week, increases the number of mature dendritic spines, and enhances the potential for metaplasticity for 24 hours to 2 weeks following a single dose (Burgdorf et al., 2015). Despite positive results of rapastinel in phase 2 studies (Preskorn et al., 2015), rapastinel administration failed to separate from placebo in phase 3 trials in adults with treatment-resistant depression (Kato and Duman, 2020). Conclusions from the phase 3 trials informed the need for dose interval schedules—specifically, appropriate dosing due to the *U*-shaped dose response—which were applicable to other members of the stinel class. This has led to further development of second- and third-generation NMDAR PAMs currently in clinical trials. Zelquistinel was shown to enhance the magnitude of LTP in rodents, which was sustained for at least 1 week following a single administration (Burgdorf et al., 2022). Evidence indicates that therapeutic effects of these compounds are not explained by the ODP model but instead are more likely to be explained by EDP, which suggests the possibility that optimal dose on a *U*-shaped response curve or frequency of dosing may not have been achieved in prior clinical studies. Zelquistinel has begun phase 2 studies (ClinicalTrials.gov identifier: NCT06547489) as a depression treatment with once-weekly administration, and the results will be proof of principle that intermittent dosing-induced plasticity using EDP is important for the therapeutic effects of rapid-acting antidepressants.

## Application and assessment of EDP pharmacology

Within the EDP model, the drug is cleared before the full effects are observed, thereby diverging from relationships characterized during traditional dose optimization (Table 1). Targeting ODP outcomes with plastogens via sustained receptor occupancy may result in few or no durable benefits while increasing side effect burden. Chronic exposure (rather than intermittent) dosing schedules may result in the premature termination of efforts toward the advancement of investigational therapeutic agents. In EDP, dosing frequency can likely be decreased (Figure 1(b)) because the therapeutic effects of the drug are in part due to the sustained changes in synaptic strength or activation of metaplasticity processes that do not require the drug to be continuously present, unlike in ODP. Repeated intermittent dosing can open a temporal therapeutic window while minimizing drug exposure, which may result in a reduction of drug side effects related directly to exposure levels (such as dissociation with ketamine) or an elimination of those that may occur following chronic exposure (such as urological toxicity, hepatotoxicity, and cognitive deficits; Short et al., 2018). For example, observations of ketamine, as well as other drugs targeting plasticity mechanisms, demonstrated an inverted *U*-shaped curve with impaired NMDAR activation and loss of efficacy at higher doses, suggesting a nonlinear mechanism (Brown and Gould, 2024; Brown et al., 2025b; Donello et al., 2019; Kim and Monteggia, 2020; Li et al., 2010; Zanos et al., 2016, 2023). Instead, within EDP, rapid-onset plastogens are subject to the Goldilocks effect, where the dose and dosing frequency are informed by the durability of the response. In addition to an increase in undesirable side effects, it is likely that sustained levels of drug will result in physiological insensitivity or engagement of compensatory homeostatic mechanisms to prevent further plasticity and a resultant lack of desirable drug effects. Moving forward, validated pharmacokinetic and pharmacodynamic models will need to be developed to determine optimal dosage and dose frequency.

To evaluate EDP, two types of biomarkers are needed, the first to measure the initial response or triggering event and the second to measure sustained plasticity or the capacity for plasticity. Cortical oscillations as measured by electroencephalography (EEG), both in the low (delta, theta, and alpha) and high (beta and gamma) frequencies, have been associated with changes in neural activity and numerous cognitive processes (Başar and Güntekin, 2013). Alterations in resting-state EEG patterns are associated with cortical functional connectivity and neurotransmission deficits (Anderson and Perone, 2018). Gamma oscillations play a key role in learning and memory function, and alterations in gamma oscillations are evident in neurodegenerative and neuropsychiatric diseases (e.g., MDD; Strüber and Herrmann, 2020) and may act as a biomarker useful in the treatment of mood disorders. In both preclinical and clinical studies, acute administration of ketamine results in increases in gamma oscillations (Hiyoshi et al., 2014; Hong et al., 2010; Medeiros et al., 2023; Muthukumaraswamy et al., 2015). These ketamine-induced increases in gamma power are predicted to be driven by preferential ketamine inhibition of GABAergic interneurons leading to disinhibition and resultant cortical excitation (Adam et al., 2024), though the (*2R,6R*)-HNK metabolite of ketamine also increases gamma oscillations without NMDAR inhibition (Raja et al., 2024; Zanos et al., 2016). It has been found that an increase in gamma power postinfusion predicted antidepressant response to ketamine in a study design where ketamine infusion was followed a week later by repeated administration of ketamine thrice weekly over a period of 2 weeks (De la Salle et al., 2022). This finding suggests that gamma oscillations may be a promising biomarker to predict response among responders and nonresponders. EEG rhythmical oscillations may be an appropriate biomarker to measure the rapid but transient initial event that is driven by a direct pharmacokinetic-pharmacodynamic relationship, which precedes the long-lasting effects. Importantly, these outcomes will measure the triggering event, which at peak concentrations (*C*_max_) will be soon after drug administration and prior to therapeutic effects in drugs with short half-lives. For example, with ketamine, *C*_max_ is typically achieved at the end of or shortly following an infusion (Zanos et al., 2018).

Measurements of plasticity could target either persistent changes in synaptic strength or the capacity of synapses to respond more favorably to plasticity-inducing stimuli. As an example of the former, evoked responses prior to and following ketamine infusions can measure the strength of circuits. For example, patients responsive to ketamine treatment showed an increased stimulus-evoked cortical excitability, as measured by magnetoencephalography, compared with nonresponders 6–7 hours after ketamine administration (Cornwell et al., 2012). Similar to preclinical methods to generate LTP, defined stimulation frequencies in vivo can result in synaptic potentiation. An example of this is utilizing transcranial magnetic stimulation (TMS)—evoked responses. It has been shown that D-cycloserine, an NMDAR partial agonist at the low doses used, can rescue TMS motor plasticity in MDD. This effect was not observed the day of administration but rather 24 hours later (Cole et al., 2021), which is indicative of EDP processes. Despite these possibilities, considering the novelty of EDP in clinical practice and the increasing promise of plastogens in CNS disease, further improvements in biomarkers will need to be developed to measure the immediate and sustained effects of these compounds.

## Conclusion

Rapid-onset plastogens may trigger rapid, durable, and safe enhancement of synaptic function, but their successful application likely requires intermittent dosing and pharmacological parameters that are typically considered undesirable in ODP models, such as a short half-life. Given the inability of the ODP model to explain the rapid and sustained therapeutic effects, neuroplastogens and psychoplastogens should be considered as operating within EDP. In this model, traditional pharmacokinetics (e.g., *C*_max_, half-life, elimination time) of the compound itself are not helpful in either characterizing or optimizing the biological effects or network changes (i.e., sustained pharmacodynamics) of the compound.

Targeting traditionally desirable pharmacokinetic properties of compounds for drug development, such as long-term receptor occupancy, may be less relevant, if not counterproductive. Novel, scientifically informed, and validated pharmacokinetic and pharmacodynamic models will need to be developed to determine optimal dosage and dose frequency. Regulating agencies may need to develop updated guidance for clinical studies of drugs engaging EDP mechanisms.

The principles of EDP we have discussed are also relevant to the administration of neuromodulation therapies and the application of the combination of pharmacotherapy and neuromodulation. Antidepressant outcomes of electroconvulsive therapy and other forms of neuromodulation have been linked to nonselective, long-lasting alterations in synaptic transmission that then promote functional and structural plasticity (Chen et al., 2023; Conway and Xiong, 2018; Deng et al., 2024; Dukart et al., 2014; Nitsche et al., 2003; Stefan et al., 2002). The findings we discussed, which show that rapid-acting antidepressants engage processes to modulate the interval, direction, or magnitude of glutamatergic synaptic plasticity events, suggest the possibility that plastogen treatment time-appropriately administered prior to neuromodulation will engage metaplastic mechanisms and enhance both the timing of onset and the sustained therapeutic actions of neuromodulation.

EDP and psychotherapy can work cooperatively to maximize the therapeutic effect for patients. Plastogens, acting through EDP, may exert either direct effects on plasticity or effects via changes in metaplasticity that will allow the brain to be more responsive to different forms of therapy, including psychotherapy and cognitive behavioral therapy. Randomized clinical trial data demonstrate that pairing a single injection of ketamine with a targeted behavioral intervention prolongs antidepressant response relative to either intervention alone, supporting the hypothesis that ketamine-induced neuroplasticity may enhance the efficacy of adjunctive psychotherapeutic approaches (Price et al., 2022). A similar double-blind, placebo-controlled study is currently underway to test the efficacy of pairing of the NMDAR PAM apimostinel with computer-assisted cognitive therapy (ClinicalTrials.gov identifier: NCT06400121). A systematic review of studies investigating the combination of psychotherapy with ketamine suggested benefit, but further investigation is needed to clarify the benefit of adjunctive psychotherapeutic approaches over plastogens alone (Kew et al., 2023). Plastogens, through EDP, may improve the capacity of the brain to change, and psychotherapy may help shape those changes into more meaningful behavioral and cognitive outcomes. Importantly, the optimal timing and sequencing of psychotherapy relative to plastogen administration requires future careful investigation to determine when interventions are most effective.

In addition to the treatment of depression, plastogens administered via EDP have substantial potential applications in other CNS diseases, including other psychiatric conditions, drug addictions, and neurodevelopmental and neurodegenerative disorders in which synaptic dysfunction and disruption of normal network activity are common underlying pathologies. Consistent with this, emerging research is exploring the role of plastogens in the treatment of posttraumatic stress disorder, anxiety disorders, and substance use disorders, among others (Feulner et al., 2023; Holze et al., 2024; Kwan et al., 2024; Modlin et al., 2025; Whittaker et al., 2021). Therapies that enhance neuroplasticity, like plastogens, may be broadly effective across multiple CNS disorders because impaired neural signaling and network dysfunction are shared mechanisms of disease.
